# Microwave-Assisted Maleation of Coconut Husk Nanolignin: Structure–Property Relationships Governed by Degree of Esterification

**DOI:** 10.3390/ijms27135950

**Published:** 2026-07-02

**Authors:** Wissawat Sakulsaknimitr, Pornpen Atorngitjawat

**Affiliations:** 1 Department of Fundamental Science and Physical Education, Faculty of Science, Kasetsart University, Sriracha Campus, Chonburi 20230, Thailand; wissawat.s@ku.th; 2 Department of Chemistry, Faculty of Science, Burapha University, Chonburi 20131, Thailand

**Keywords:** nanolignin, microwave-assisted synthesis, sustainable biomaterials, thermo-oxidative stability

## Abstract

Coconut husk lignin was fractionated using ethanol to obtain nanolignin fractions with distinct physicochemical properties. Among the fractions, CNF1 exhibited the most favorable combination of particle size, thermal stability, and antibacterial activity and was selected for further modification. Microwave-assisted esterification of CNF1 with maleic anhydride was performed under various reaction temperatures and lignin-to-maleic anhydride ratios. Structural modification was confirmed by ATR-FTIR spectroscopy through the appearance of ester carbonyl groups and an increase in the degree of esterification, which reached its highest value at 180 °C and a lignin-to-maleic anhydride ratio of 1:10. TEM analysis revealed that maleation increased nanoparticle size, whereas WAXD demonstrated that both native and modified lignins retained predominantly amorphous structures. Antioxidant activity decreased with increasing esterification due to the reduction of phenolic hydroxyl groups. Thermal analysis showed that esterification altered the degradation behavior of lignin, while thermo-oxidative stability measurements indicated improved oxidation resistance for highly esterified samples. The 10MA180 sample exhibited the highest thermo-oxidative stability, with a T_2_,O_2_ value of 12.55 min, a residual mass of 84.90%, and the lowest weight loss after 60 min oxidation. These findings demonstrate that microwave-assisted maleation effectively tailors the structure and functional properties of nanolignin for sustainable bio-based material applications.

## 1. Introduction

Nowadays, due to mounting environmental concerns and growing interest in nature-conservation principles, there is an increasing drive to reduce reliance on non-renewable raw materials and to adopt more cost-effective, sustainable feedstocks. Large volumes of agricultural waste and industrial by-products are generated annually. According to the Intergovernmental Panel on Climate Change (IPCC), total global anthropogenic greenhouse gas emissions reached approximately 59 Gt CO_2_-equivalent in 2019, underscoring the need for sustainable biomass valorization strategies [1]. In many regions, agricultural residues are still disposed of through open burning or inefficient combustion, generating greenhouse gas emissions and contributing to air pollution. Therefore, the valorization of lignocellulosic agricultural wastes into high-value bio-based materials has emerged as an important strategy for reducing waste-related emissions, promoting carbon utilization, and supporting the development of a circular bioeconomy [1,2,3]. Despite their abundance, many agricultural residues are still used only as low-grade industrial fuels or basic bio-fertilizers, and their potential as renewable feedstocks for high-value materials remains largely underutilized. Among the various agricultural by-products, coconut husks represent a promising lignocellulosic resource due to their abundance, low cost, and high carbon content [4,5]. Traditionally, these waste materials have been disposed of through incineration as low-grade industrial fuels or converted into bio-fertilizers. However, such practices offer limited value addition and may still contribute to carbon emissions and environmental pollution. Consequently, transforming these residues into high-value biopolymers or nanomaterials provides an attractive strategy for waste valorization and circular bioeconomy development [6]. Importantly, coconut husks are rich in lignocellulosic polymers, primarily cellulose, hemicellulose, and lignin. Previous studies have reported that coconut husks typically contain approximately 29% cellulose, 20–30% hemicellulose, and 44% lignin [7]. Lignin itself is a complex aromatic biopolymer built from phenylpropanoid units such as p-coumaryl, coniferyl, and sinapyl alcohols. It is highly branched and cross-linked, forming a heterogeneous three-dimensional polymeric network within the plant cell wall [8,9]. The polymer is highly resistant to degradation and confers structural rigidity, hydrophobicity, and thermal stability to biomass. Because of these properties, lignin is increasingly viewed as a valuable renewable feedstock rather than simply a burning fuel. Extracted lignin has found applications in bio-composite materials, chemical precursors, functional additives, UV-absorbing agents, and nanomaterials.

Owing to its high density of phenolic and aliphatic hydroxyl groups, lignin possesses considerable potential for chemical functionalization and valorization into value-added bio-based materials. Among various modification strategies, esterification with cyclic anhydrides has attracted considerable attention because it can alter lignin polarity, improve compatibility with polymer matrices, and introduce reactive functional groups for further crosslinking reactions [10,11]. In particular, maleic anhydride is an attractive esterifying agent because it can graft ester-linked maleate moieties onto lignin, thereby introducing carbonyl groups and unsaturated C=C bonds into the lignin structure. These newly introduced functionalities can participate in subsequent polymerization or copolymerization reactions, expanding the applicability of lignin in thermosets, coatings, composites, adhesives, and bio-based resin systems [12,13,14]. Recent studies have demonstrated that maleated technical lignin can act as a reactive platform for the preparation of lignin-based thermosets and biocomposites with improved material performance and potential degradability [12].

Microwave-assisted synthesis has emerged as an efficient alternative to conventional heating for biomass and polymer modification because microwave irradiation provides rapid volumetric heating, enhances molecular mobility, improves reaction efficiency, and can significantly reduce reaction time [15,16]. These advantages are particularly relevant for lignin esterification, where efficient activation of hydroxyl groups and uniform energy transfer can promote the formation of ester bonds under shorter reaction conditions. Structural changes following esterification are commonly confirmed by Fourier transform infrared spectroscopy (FTIR), especially through the appearance or enhancement of carbonyl stretching bands associated with ester formation, while aromatic skeletal vibrations can be used as internal reference bands for evaluating relative structural changes [17]. In addition to altering the chemical structure, lignin modification may also affect particle morphology, molecular packing, antioxidant activity, thermal behavior, and interfacial interactions with surrounding materials. Furthermore, the structural organization and chemical functionality of lignin play a crucial role in governing interfacial adhesion and compatibility with polymeric matrices. The type, density, and distribution of functional groups influence intermolecular interactions, surface energy, dispersion behavior, and stress transfer efficiency at the lignin–polymer interface. Consequently, tailored lignin structures can improve adhesion performance and facilitate the development of sustainable materials with tunable physicochemical properties, including nanocomposites, coatings, thermosets, adhesives, and hybrid bio-based systems. Therefore, controlling lignin architecture through chemical modification represents an important strategy for expanding the utilization of lignin in advanced functional materials [18,19]. Lignin nanoparticles are typically amorphous aromatic materials with morphology and functionality strongly influenced by extraction, fractionation, and modification conditions [20,21]. Moreover, lignin is recognized as a natural antioxidant, mainly due to its phenolic hydroxyl groups and electron-donating aromatic structure; therefore, esterification may reduce antioxidant activity by consuming free hydroxyl groups [22,23,24]. Thermal and thermo-oxidative analyses are also important for evaluating the carbonization behavior, char-forming ability, and oxidative stability of lignin-based materials, which are critical for applications in thermosets, coatings, composites, and high-performance bio-based resins [4,13,14].

However, despite these advances, limited information is available regarding the structure–property relationships of microwave-assisted maleated coconut husk nanolignin. In particular, the influence of the degree of esterification on morphological characteristics, antioxidant activity, thermal stability, and thermo-oxidative stability has not yet been systematically investigated. Therefore, in this study, fractionated coconut husk nanolignin was modified with maleic anhydride under microwave irradiation. The structural modification was characterized using FTIR spectroscopy and relative degree of esterification analysis. Morphology and structural properties were examined by transmission electron microscopy (TEM) and wide-angle X-ray diffraction (WAXD), respectively. Antioxidant activity was determined using the Ferric Reducing Antioxidant Power (FRAP) assay, while thermal and thermo-oxidative stability were investigated to evaluate carbonization behavior and oxidative resistance. The relationships between the degree of esterification and the resulting morphology, antioxidant activity, thermal stability, and thermo-oxidative stability were systematically investigated to elucidate how maleation governs the properties of microwave-assisted maleated coconut husk nanolignin.

## 2. Results and Discussion

### 2.1. Characterization of Lignin

#### 2.1.1. Chemical Characteristics of Nano-Lignin

**Figure 1:**
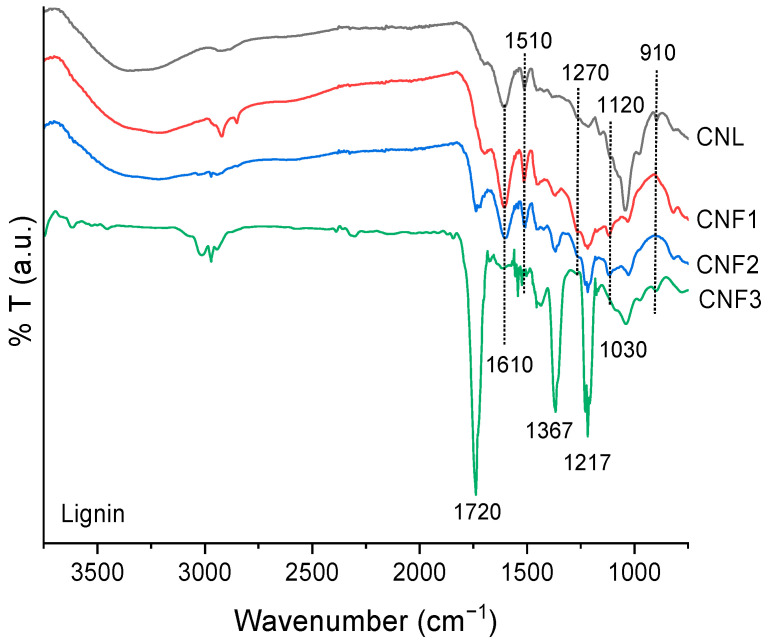
ATR-FTIR spectra of lignin fractions CNL, CNF1, CNF2, and CNF3.

The FTIR spectra of coconut husk lignin (CNL) and its fractions (CNF1–CNF3) are displayed in Figure 1. The spectra exhibit the characteristic absorption bands of lignin, confirming the preservation of the aromatic polymeric structure after extraction and fractionation. The prominent bands at approximately 1610 and 1510 cm^−1^ correspond to aromatic skeletal vibrations (C=C stretching) of the lignin phenylpropane units, indicating the presence of a well-defined lignin backbone [17,25]. A distinct absorption at ~1270 cm^−1^, assigned to guaiacyl (G) ring breathing with C–O stretching, is clearly observed in all samples, suggesting that coconut lignin is predominantly composed of guaiacyl units [26,27]. In contrast, the relatively weak or nearly absent band at ~1120 cm^−1^, typically attributed to syringyl (S) units, indicates a low syringyl content. The band at ~1217 cm^−1^ is associated with aryl–O stretching vibrations, while the peak at ~1030 cm^−1^ corresponds to C–O stretching in primary alcohols and ether linkages. Additionally, the absorption at ~1720 cm^−1^, particularly pronounced in CNF3, is assigned to unconjugated carbonyl groups (C=O) arising from ester or oxidized side chains, suggesting partial oxidation or structural modification during processing [8,28]. The band at ~1367 cm^−1^ is attributed to aliphatic C–H deformation and phenolic OH bending, although it is not specific to syringyl units. The FTIR results indicate that coconut core lignin is a guaiacyl-rich lignin with minor contributions from other functional groups, consistent with typical lignin structures derived from non-woody biomass and agricultural residues.

#### 2.1.2. Particle Size of Lignin

**Figure 2:**
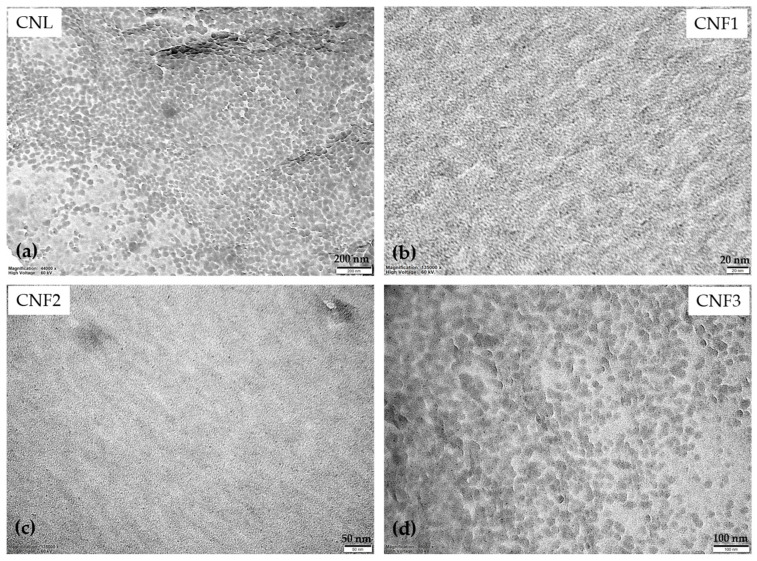
TEM micrographs of lignin fractions: (**a**) CNL, (**b**) CNF1, (**c**) CNF2, and (**d**) CNF3.

The morphology and particle size distribution of lignin fractions (CNL, CNF1, CNF2, and CNF3) were investigated by TEM, as shown in Figure 2. All fractions consisted predominantly of spherical nanoparticles with relatively uniform dispersion, although partial aggregation was observed in some regions where adjacent particles formed irregular clusters. Such aggregation is commonly observed in lignin nanoparticles and is mainly attributed to intermolecular hydrogen bonding, hydrophobic interactions, and π–π stacking between aromatic lignin molecules [29,30].

**Figure 3:**
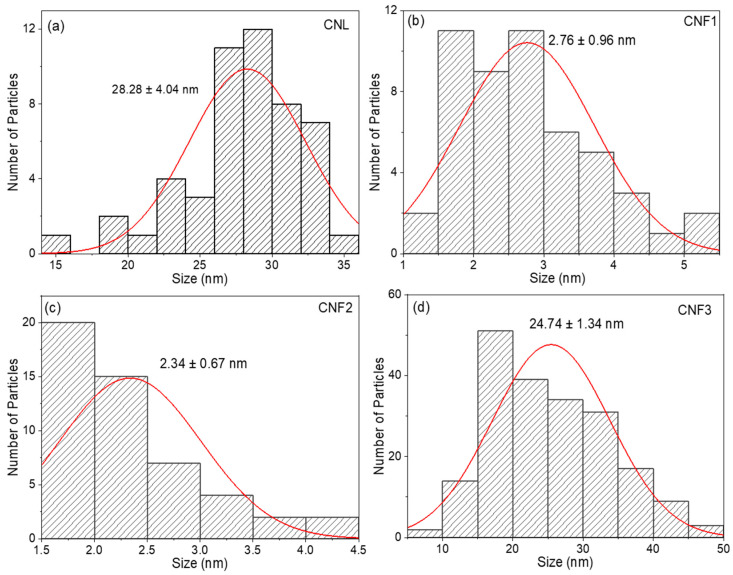
Particle size distributions of (**a**) CNL, (**b**) CNF1, (**c**) CNF2, and (**d**) CNF3 determined from TEM images.

The particle size distributions obtained from TEM analysis are presented in Figure 3. The average particle diameters of CNL, CNF1, CNF2, and CNF3 were 28.28 ± 4.04, 2.76 ± 0.96, 2.34 ± 0.67, and 24.74 ± 1.34 nm, respectively. Among the fractions, CNF1 and CNF2 exhibited remarkably small particle sizes (<3 nm), indicating that ethanol fractionation preferentially enriched highly soluble, low-molecular-weight lignin fractions. Previous studies have shown that ethanol fractionation selectively isolates lignin with lower molecular weight, lower degrees of condensation, and reduced intermolecular association, resulting in smaller and more homogeneous nanoparticles [31,32]. Accordingly, CNF1 and CNF2 likely consisted of highly dispersed lignin fragments with limited aggregation. In contrast, CNL exhibited the largest average particle size and the broadest size distribution, indicating a heterogeneous mixture of lignin populations. The average particle size of CNL was comparable to that of CNF3, suggesting that larger lignin particles contributed more substantially to the morphology of the unfractionated sample. The relatively large particle size of CNF3 (24.74 ± 1.34 nm) further suggests the presence of higher-molecular-weight lignin fractions with stronger intermolecular interactions, promoting particle growth through aromatic stacking and hydrogen bonding [33]. The narrower particle size distribution of CNF3 compared with CNL indicates that ethanol fractionation effectively separated lignin fractions according to their physicochemical characteristics.

Despite these differences, all fractions remained within the nanometer range (approximately 2–30 nm), confirming the successful production of nanolignin. The particle sizes observed in this study are considerably smaller than those typically reported for lignin nanoparticles prepared by conventional nanoprecipitation or solvent-shifting methods (50–300 nm) [34]. This exceptionally small particle size is attributed to the extraction and ethanol fractionation procedures, which enriched highly soluble, low-molecular-weight lignin fractions with reduced intermolecular association. Furthermore, particle size was determined directly from representative TEM micrographs by measuring individual primary particles rather than aggregated structures. Consequently, the reported values represent the dimensions of highly dispersed primary nanolignin particles, which are substantially smaller than the aggregate sizes commonly reported for lignin nanoparticles prepared by conventional methods. The reduced particle size is expected to increase the specific surface area of the particles, thereby enhancing the accessibility of phenolic hydroxyl groups and potentially improving antioxidant performance [35].

#### 2.1.3. Antibacterial Activity Test

The antibacterial activity of the four nano-lignin extracts against Gram-positive *Staphylococcus aureus* and Gram-negative *Escherichia coli* is presented in Table 1.

All nanolignin fractions exhibited excellent antibacterial activity against *S. aureus*, with antibacterial activity efficiency (AAE) values of 100%. In contrast, no detectable antibacterial activity was observed against *E. coli*, and all samples exhibited AAE values of 0%. This difference can be attributed to the structural characteristics of Gram-negative bacteria. *E. coli* possesses an outer membrane containing lipopolysaccharides, which acts as an effective permeability barrier and restricts the penetration of lignin-derived phenolic compounds. In contrast, *S. aureus* lacks this outer membrane and is therefore generally more susceptible to lignin-derived antimicrobial agents [36,37,38]. Among the tested samples, CNF1 and CNF2 exhibited the strongest antibacterial performance against *S. aureus*, which may be associated with their smaller particle size and greater surface area, facilitating more effective interactions with bacterial cells.

#### 2.1.4. Thermal Stability of Lignin Fractions

**Figure 4:**
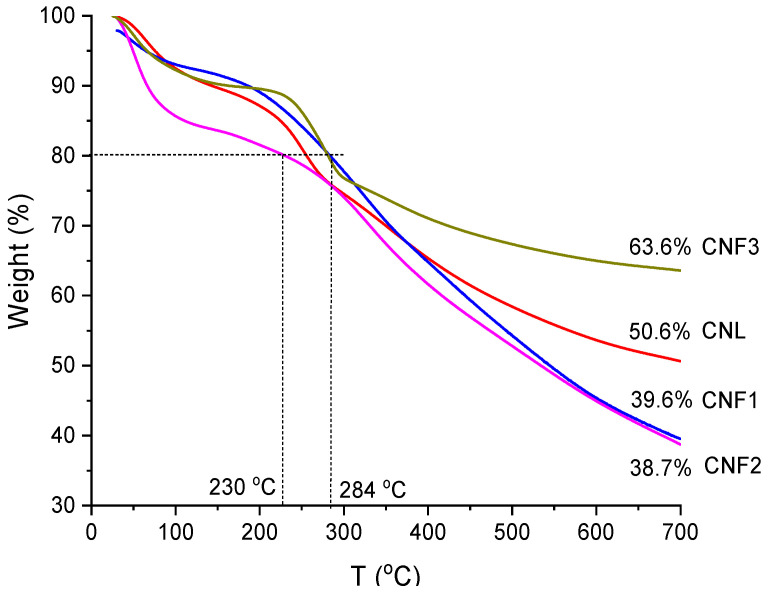
Thermal stability of lignin fractions under N_2_ atmosphere.

The thermal stability of CNL, CNF1, CNF2, and CNF3 was evaluated by TGA under a nitrogen atmosphere, and the corresponding thermograms are presented in Figure 4. All samples exhibited the typical three-stage thermal degradation behavior of lignin. The first stage below approximately 150 °C was attributed to the evaporation of moisture and low-molecular-weight volatiles. The second stage (approximately 200–400 °C) corresponded to the main decomposition of the lignin structure through cleavage of ether linkages, side chains, and oxygen-containing functional groups. The final stage at higher temperatures was associated with carbonization and char formation resulting from the condensation of aromatic structures [39,40].

As indicated by the dashed lines in Figure 4, the temperatures corresponding to 20% weight loss (80% residual mass) were approximately 256, 284, 230, and 281 °C for CNL, CNF1, CNF2, and CNF3, respectively. Among the four fractions, CNF1 exhibited the highest thermal stability, whereas CNF2 showed the lowest resistance to thermal degradation. The superior thermal stability of CNF1 is attributed to the enrichment of relatively homogeneous, low-molecular-weight lignin fractions with fewer thermally labile components following ethanol fractionation [28]. In contrast, the lower thermal stability of CNF2 suggests a greater abundance of oxygen-containing functional groups and structurally weaker lignin fragments that undergo thermal cleavage more readily [40]. A clear difference was also observed in the char residue at 700 °C. CNF3 exhibited the highest residual mass (~63.6%), considerably higher than those of CNL (~50.6%), CNF1 (~39.6%), and CNF2 (~38.7%). The high char yield indicates that CNF3 contains a greater proportion of condensed aromatic structures and higher-molecular-weight lignin components, which promote carbonization during pyrolysis and enhance thermal resistance at elevated temperatures [41,42]. Considering the overall physicochemical and biological properties, CNF1 exhibited the most favorable combination of ethanol solubility, nanoscale particle size, thermal stability, and antibacterial activity. Therefore, CNF1 was selected as the precursor for subsequent microwave-assisted maleation to further enhance its functionality for advanced bio-based materials.

### 2.2. Optimization of Microwave-Assisted Synthesis of Maleated Lignin

The esterification of CNF1 with maleic anhydride was significantly influenced by reaction temperature and reagent dosage under microwave irradiation. As shown in Table 2, the reaction at lower temperatures (80–140 °C) resulted in a low recovery of solid products, with %yields ranging between 42.95% and 52.39%. This suggests that at these temperatures, the thermal energy was insufficient to drive the esterification within the short 10-min duration. Crucially, a control experiment conducted at 160 °C without the addition of maleic anhydride resulted in a 0% yield, with no solid product recovered upon precipitation. This confirms that maleic anhydride is essential for the formation of the modified lignin product, as the reaction introduces maleate groups that decrease solubility in water, allowing the modified lignin to precipitate. However, increasing the temperature to 160 and 180 °C led to a substantial increase in %yield, with values exceeding 100% at 180 °C indicating a high degree of maleic anhydride grafting onto the lignin backbone.

### 2.3. Characterization of Maleated Lignin

#### 2.3.1. FTIR Analysis

**Figure 5:**
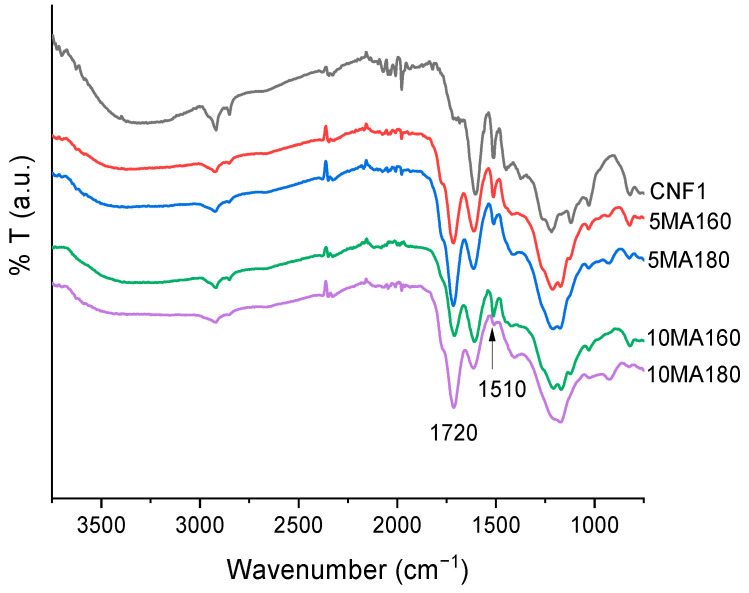
ATR-FTIR spectra of CNF1, 5MA160, 5MA180, 10MA160 and 10MA180.

The ATR-FTIR spectra of 5MA160, 5MA180, 10MA160 and 10MA180 (used as examples) are shown in Figure 5. The chemical structural changes of CNF1 after maleation were investigated. The spectrum of native CNF1 exhibited the characteristic absorption bands of lignin. A broad band centered at approximately 3400 cm^−1^ was assigned to the stretching vibration of hydroxyl groups (O–H) originating from phenolic and aliphatic hydroxyl functionalities. The absorption bands near 2930 and 2840 cm^−1^ were attributed to the asymmetric and symmetric stretching vibrations of aliphatic C–H groups. Characteristic aromatic skeletal vibrations of lignin were observed at approximately 1600, 1510, and 1425 cm^−1^, corresponding to the aromatic phenylpropane units that constitute the lignin macromolecule [13,17].

After maleation treatment, significant changes were observed in the FTIR spectra. The most prominent feature was the appearance and progressive increase in intensity of the absorption band located at approximately 1710–1735 cm^−1^, corresponding to the stretching vibration of ester carbonyl groups (C=O). The increasing intensity of this band with increasing maleic anhydride content and reaction temperature indicates the successful grafting of maleate moieties onto the lignin structure through esterification reactions between maleic anhydride and lignin hydroxyl groups. Similar spectral changes have been reported for maleated lignin and esterified lignocellulosic materials [43,44].

Simultaneously, the intensity of the broad hydroxyl absorption band around 3400 cm^−1^ gradually decreased after modification, suggesting the consumption of free hydroxyl groups during ester bond formation. Furthermore, enhanced absorption in the region of 1150–1250 cm^−1^ was observed, which can be attributed to C–O stretching vibrations of ester linkages. These observations provide additional evidence for the successful esterification of lignin. Among the modified samples, 10MA180 exhibited the strongest carbonyl absorption and the greatest reduction in hydroxyl intensity, indicating the highest degree of esterification. In contrast, 5MA160 showed a less pronounced carbonyl band, suggesting a lower extent of modification under milder reaction conditions. These results are in agreement with the degree of esterification values determined independently and demonstrate that both maleic anhydride loading and reaction temperature significantly influenced the efficiency of lignin modification.

Although the FTIR spectra, the relative degree of esterification analysis, and the associated changes in physicochemical properties collectively provide strong evidence for successful esterification, it should be acknowledged that FTIR alone cannot unequivocally distinguish covalently bonded maleate groups from all possible strongly associated residual maleate species. Nevertheless, the extensive washing procedure employed after the reaction, the systematic dependence of the relative degree of esterification on reaction temperature and maleic anhydride loading, together with the consistent changes observed in particle morphology, antioxidant activity, thermal stability, and thermo-oxidative stability, collectively support the occurrence of chemical modification rather than simple physical adsorption of maleate species. Further confirmation using quantitative techniques such as ^13^C NMR spectroscopy, saponification analysis, or acid–base back-titration will be considered in future studies to provide more direct evidence of covalent ester bond formation.

#### 2.3.2. Effect of Temperature on Product Yield and Degree of Esterification

**Figure 6:**
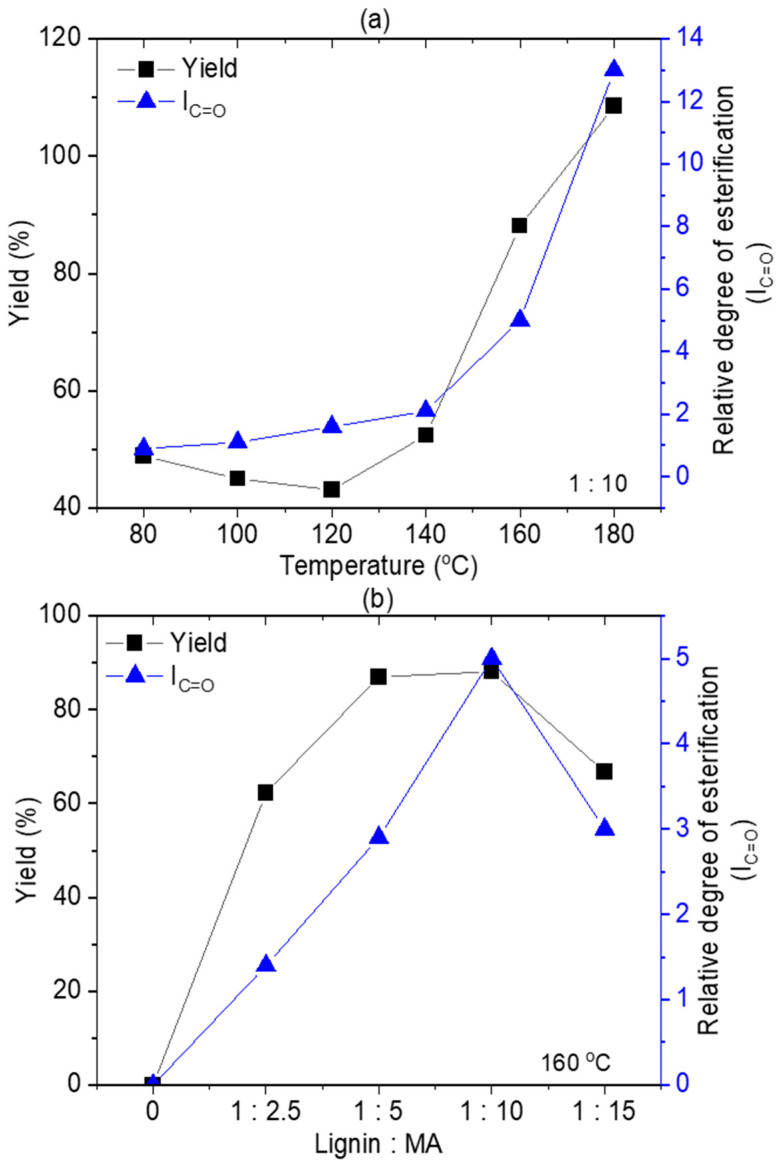
Relative degree of esterification (I_C=O_) as a function of (**a**) reaction temperature and (**b**) lignin and maleic anhydride ratio.

The effects of reaction temperature and maleic anhydride loading on product yield and esterification efficiency are presented in Figure 6. The degree of esterification was estimated from the relative intensity of the carbonyl absorption band (I_C=O_) obtained from FTIR analysis, which is widely used as a relative indicator of ester group formation in modified lignin [43]. As shown in Figure 6a, increasing the reaction temperature from 160 to 180 °C resulted in a significant increase in both product yield and I_C=O_ values. At a maleic anhydride-to-lignin ratio of 5:1, the product yield increased from approximately 49% at 160 °C to 88% at 180 °C, while the I_C=O_ value increased from approximately 1.0 to 5.1. Similarly, at a ratio of 10:1, the yield increased from approximately 43% to 108%, accompanied by an increase in I_C=O_ from approximately 1.3 to 12.8. These results indicate that elevated reaction temperatures promote esterification reactions between lignin hydroxyl groups and maleic anhydride, leading to greater incorporation of maleate functionalities into the lignin structure. The increase in esterification efficiency at higher temperatures can be attributed to enhanced molecular mobility, improved diffusion of maleic anhydride into the lignin matrix, and increased reaction kinetics. Higher temperatures facilitate the opening of the anhydride ring and accelerate the formation of ester linkages with phenolic and aliphatic hydroxyl groups present in lignin [43,45]. Consequently, the increase in the ester carbonyl absorption band is consistent with a higher extent of lignin modification under these reaction conditions.

The influence of maleic anhydride loading is further illustrated in Figure 6b. Increasing the maleic anhydride ratio from 0 to 10 resulted in a substantial increase in both product yield and I_C=O_ values. The yield increased sharply from the untreated sample to approximately 87–89% at ratios of 5 and 10, whereas the I_C=O_ value increased continuously and reached a maximum at the 10:1 ratio. This trend suggests that a greater availability of maleic anhydride molecules increases the probability of reaction with lignin hydroxyl groups, thereby enhancing the degree of esterification. Notably, the increase in I_C=O_ was more pronounced than the increase in product yield, indicating that chemical modification continued even when the overall product recovery approached a plateau. The highest esterification efficiency was achieved at 180 °C with a maleic anhydride-to-lignin ratio of 10:1, as evidenced by the maximum I_C=O_ value. This finding suggests that both elevated temperature and excess maleic anhydride are critical factors for maximizing the extent of lignin esterification. The product yield exceeding 100% for sample 10MA180 resulted from the calculation method based on the initial lignin mass. Esterification with maleic anhydride introduced additional covalently bound maleate groups into the lignin structure, increasing the molecular weight of the modified lignin and consequently the recovered product mass. The exceptionally high I_C=O_ value obtained for 10MA180 is consistent with a greater extent of lignin modification under these reaction conditions. Similar mass increases have been reported for lignin esterification reactions involving cyclic anhydrides, where covalent incorporation of anhydride-derived groups contributes directly to the final product weight.

#### 2.3.3. Morphological Properties

**Figure 7:**
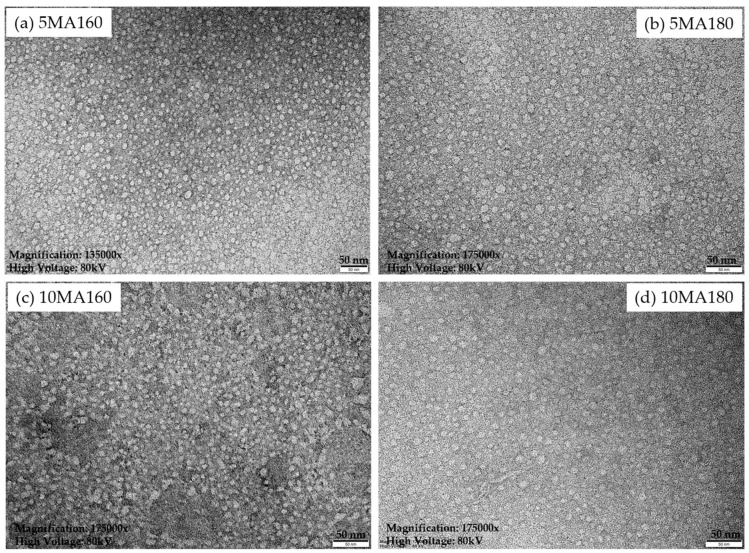
TEM micrographs of maleated lignin: (**a**) 5MA160, (**b**) 5MA180, (**c**) 10MA160, and (**d**) 10MA180.

**Figure 8:**
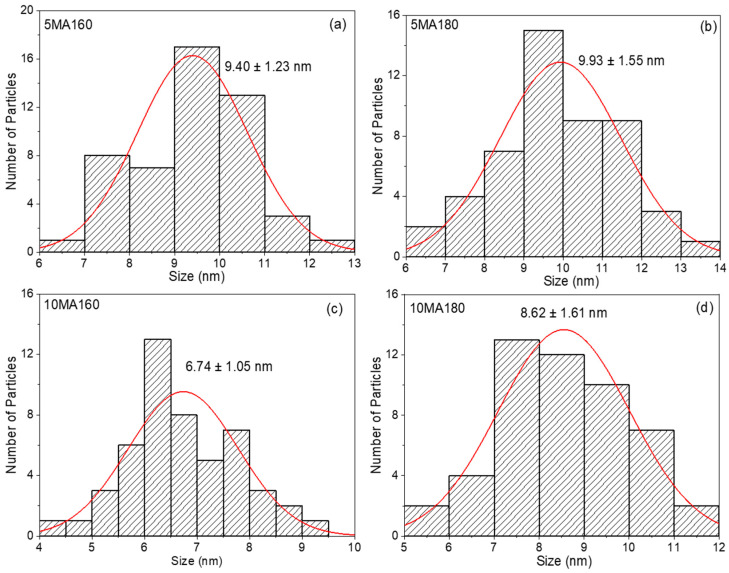
Particle size distributions of maleated lignin determined from TEM images: (**a**) 5MA160, (**b**) 5MA180, (**c**) 10MA160, and (**d**) 10MA180.

As presented in Figure 3b. The TEM micrograph of CNF1 revealed the presence of extremely fine nanoparticles with a relatively homogeneous distribution and an average particle size of 2.76 ± 0.96 nm. Following maleation treatment, significant morphological changes were observed. All modified samples exhibited larger and more distinct spherical nanoparticles compared with CNF1, as shown in Figure 7 and Figure 8. The average particle size increased to 9.40 ± 1.23 nm for 5MA160 and 9.93 ± 1.55 nm for 5MA180. This increase suggests that esterification with maleic anhydride altered the intermolecular interactions of lignin molecules and promoted the formation of larger self-assembled structures. The introduction of maleate groups onto the lignin backbone increases steric hindrance and reduces the density of free hydroxyl groups available for hydrogen bonding, resulting in a rearrangement of molecular packing and aggregation behavior [11,43]. Interestingly, increasing the maleic anhydride loading from 1:5 to 1:10 resulted in different particle size trends depending on the reaction temperature. The 10MA160 sample exhibited the smallest particle size among the modified lignins (6.74 ± 1.05 nm), whereas 10MA180 showed an intermediate size of 8.62 ± 1.61 nm. The reduction observed for 10MA160 may indicate that a higher degree of substitution increased electrosteric repulsion between modified lignin chains, thereby limiting particle growth and suppressing aggregation. Similar behavior has been reported for chemically modified lignin nanoparticles, where increased surface functionalization improves colloidal stabilization and restricts particle coalescence [29]. The particle size distributions further confirmed these morphological differences. CNF1 exhibited a narrow distribution centered around 2–3 nm, whereas the maleated samples showed broader distributions within the range of approximately 5–13 nm. The broader size distribution indicates increased heterogeneity in the self-assembly behavior of modified lignin molecules after esterification. Nevertheless, all samples remained within the nanometer scale, demonstrating that the maleation process preserved the nanolignin character while modifying the particle architecture.

The TEM results correlated well with the FTIR and degree of esterification analyses. Samples exhibiting higher esterification degrees generally showed larger nanoparticle dimensions than native lignin, confirming that the introduction of maleate functionalities altered intermolecular interactions and self-assembly behavior.

#### 2.3.4. Structural Properties

**Figure 9:**
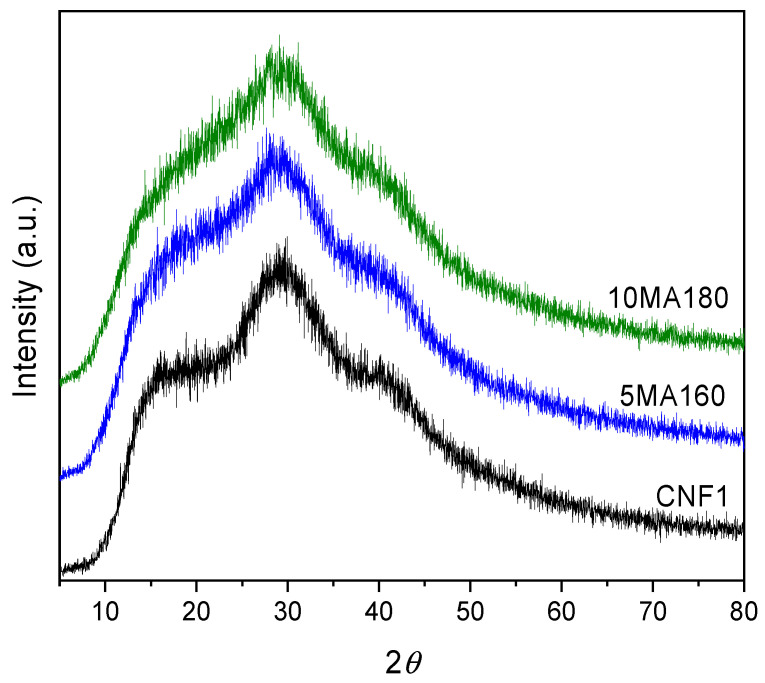
Wide-angle X-ray diffraction (WAXD) patterns of lignin and maleated lignins.

WAXD patterns of CNF1, 5MA160, and 10MA180 are presented in Figure 9. All samples exhibited broad diffraction halos characteristic of predominantly amorphous lignin structures. The diffraction profiles extended over a wide range of approximately 15–35° (2θ), with the maximum intensity observed near 29–30°. Such broad scattering behavior is typical of lignin and reflects the absence of long-range crystalline ordering arising from its highly irregular and crosslinked aromatic structure [11,33]. Similar amorphous diffraction features have been reported for various technical and nanostructured lignins, although the exact position of the intensity maximum may vary depending on lignin source, extraction method, molecular packing, and supramolecular organization [11,33,46]. Following maleation treatment, the overall diffraction profiles remained largely unchanged, indicating that esterification did not induce the formation of new crystalline phases. The slightly enhanced intensity observed for the modified samples may reflect minor changes in short-range molecular ordering associated with the introduction of maleate functionalities [33].

#### 2.3.5. Antioxidant Activity (FRAP)

**Figure 10:**
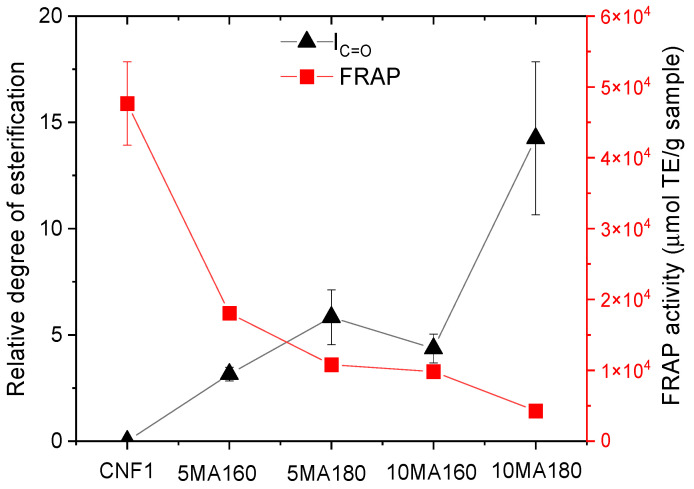
Relationship between I_C=O_ and antioxidant activity of maleated lignins.

The antioxidant activity of CNF1 and maleated samples was evaluated using the ferric reducing antioxidant power (FRAP) assay. The FRAP method measures the ability of antioxidant compounds to reduce ferric ions (Fe^3+^) to ferrous ions (Fe^2+^) through electron donation under acidic conditions [22]. As shown in Figure 10, the results demonstrated that antioxidant activity decreased markedly after esterification treatment. Native CNF1 exhibited the highest FRAP value of 47,670 ± 5888 µmol TE/g sample, indicating strong reducing power and high electron-donating capability. This high antioxidant activity was mainly attributed to the abundance of free phenolic hydroxyl groups present in the lignin structure, which are known to play an important role in radical scavenging and redox reactions [23,47]. Following maleation treatment, the FRAP activity significantly decreased with increasing degree of esterification. Samples with lower esterification degrees, such as 5MA160, still retained moderate antioxidant activity (18,060 ± 554 µmol TE/g), whereas highly esterified samples such as 10MA180 exhibited substantially lower activity (4267 ± 672 µmol TE/g). The decrease in antioxidant activity suggested that esterification consumed or blocked phenolic hydroxyl groups, thereby reducing the number of active sites available for electron transfer reactions. Similar reductions in antioxidant performance after chemical modification of lignin have been previously reported, particularly when hydroxyl functionalities are converted into ester groups [43,48].

A strong negative correlation between degree of esterification and FRAP activity was observed, with an approximate coefficient of determination of R^2^ ≈ 0.86. This relationship indicated that increasing esterification directly affected the reducing capability of the samples. Higher maleic anhydride loading and elevated reaction temperature promoted ester bond formation, leading to progressive reduction of phenolic hydroxyl content. Since phenolic hydroxyl groups are primarily responsible for hydrogen atom donation and electron transfer in lignin-based antioxidants, their reduction consequently lowered the FRAP activity [33]. The FRAP results confirmed that esterification significantly altered the antioxidant behavior of the materials. Although chemical modification may improve compatibility and reactivity in polymeric applications, it simultaneously reduced the intrinsic antioxidant properties associated with native phenolic structures. These findings suggested that balancing functionalization degree and antioxidant retention is important for developing lignin-based functional materials with both improved processability and bioactive performance. The unusually high FRAP activity of CNF1 may be associated with its extremely small particle size (2.76 ± 0.96 nm). The reduced particle size likely increased the specific surface area and exposed a greater number of redox-active phenolic sites, thereby enhancing electron transfer efficiency and ferric ion reduction capacity.

#### 2.3.6. Thermal Stability Under N_2_ Atmosphere

**Figure 11:**
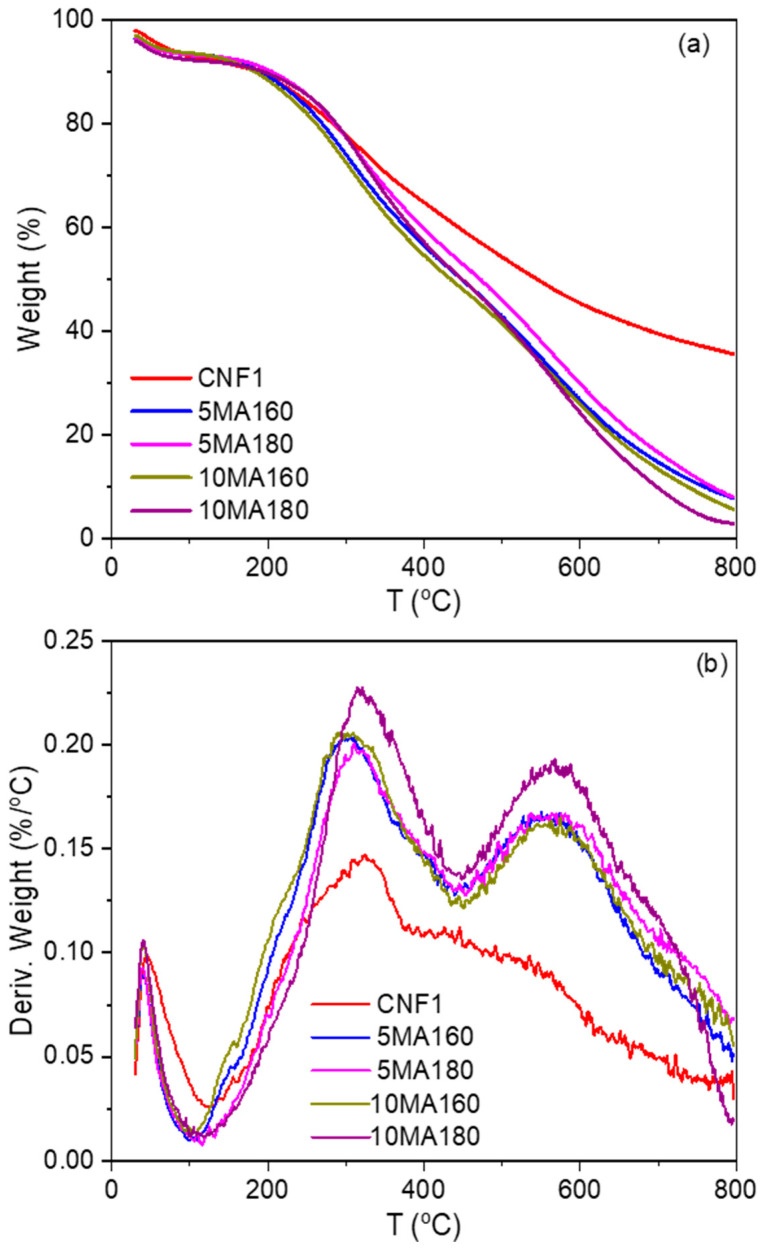
(**a**) TGA and (**b**) DTG curves of lignin and maleated lignins under N_2_ atmosphere.

The thermal stability of CNF1 and maleated lignin samples was investigated by TGA under a nitrogen atmosphere, and the corresponding TG and DTG curves are presented in Figure 11. All samples exhibited a characteristic lignin degradation profile consisting of three major stages. The initial weight loss below approximately 150 °C was attributed to the evaporation of physically adsorbed moisture and residual low-molecular-weight compounds. The principal degradation stage occurred between approximately 200 and 450 °C and was associated with the cleavage of ether linkages, decomposition of oxygen-containing functional groups, and fragmentation of the lignin macromolecular network [39,40].

The thermal stability of CNF1 and maleated lignins is summarized in Table 3. The thermal decomposition behavior of lignin is strongly influenced by its aromatic network structure, intermolecular interactions, and the presence of thermally labile functional groups [40]. The results revealed that maleation treatment significantly affected the thermal degradation characteristics of lignin. The temperature corresponding to 10% weight loss (T10%) increased from 186.22 °C for CNF1 to 205.62 °C for 5MA180, indicating improved resistance to initial thermal degradation after esterification. A similar trend was observed for 10MA180 (194.71 °C). The increase in T10% may be attributed to the reduction of free hydroxyl groups and the formation of ester linkages between lignin and maleic anhydride, which decrease moisture affinity and suppress early-stage volatilization reactions [43,44]. However, a different trend was observed for the temperature at 20% weight loss (T20%). Compared with CNF1 (283.45 °C), samples 5MA160 and 10MA160 exhibited lower T20% values of 269.36 and 261.86 °C, respectively. This reduction suggests that maleation introduced thermally labile ester linkages, which promoted degradation during the main decomposition stage. Similar effects of esterification on the thermal degradation behavior of lignin have been reported previously [31]. The maximum decomposition temperature (T_max_), corresponding to the highest degradation rate, also decreased after modification. CNF1 exhibited the highest T_max_ value (326.16 °C), whereas maleated samples showed T_max_ values between 309.56 and 320.75 °C. The reduction in T_max_ indicates that thermal scission of the modified lignin structure occurred more readily than in the native lignin. The decomposition of grafted maleate structures likely accelerated the generation of volatile fragments during the main degradation stage. Nevertheless, the sample prepared at the highest modification severity (10MA180) exhibited a relatively high T_max_ (320.75 °C), suggesting that elevated reaction temperature may have promoted additional intermolecular condensation or crosslinking reactions, partially compensating for the destabilizing effect of ester groups [48]. A marked reduction in char residue at 600 °C was observed after maleation. Native CNF1 produced a residue of 45.38%, whereas maleated samples generated only 24.22–29.86% residue. Lignin is known for its high char-forming ability due to its highly condensed aromatic structure [39]. The lower residue observed for maleated lignins indicates that esterification partially disrupted the condensed aromatic network and promoted the formation of volatile degradation products during pyrolysis. Consequently, less carbonaceous char remained after thermal decomposition. These results indicate that maleation improved the resistance to initial thermal degradation, as evidenced by increased T10% values, but simultaneously reduced the stability of the main lignin framework, resulting in lower T_max_ and reduced char formation. Among the modified samples, 10MA180 exhibited the most balanced thermal behavior, combining relatively high T10% and T_max_ values with the highest degree of esterification.

#### 2.3.7. Thermo-Oxidative Stability Under O_2_ Atmosphere

**Figure 12:**
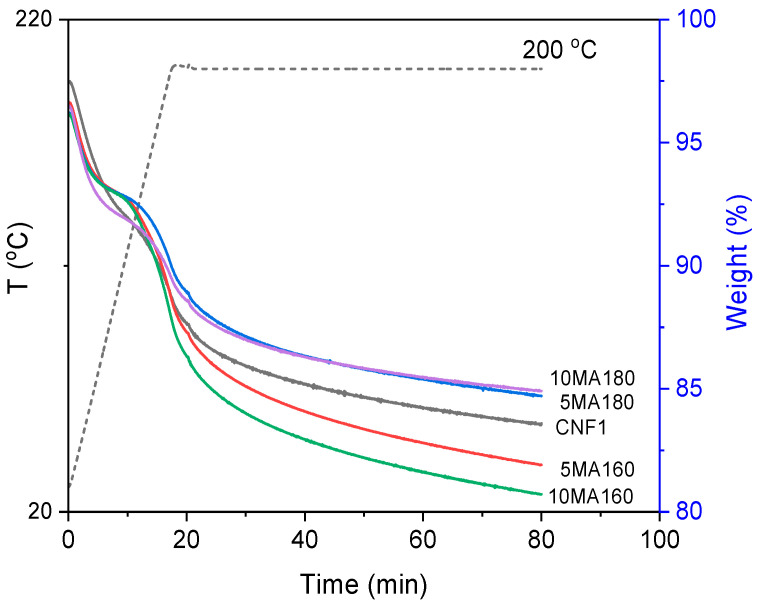
Thermo-oxidative degradation profiles of CNF1 and maleated lignins under oxygen atmosphere, demonstrating the effect of maleation on oxidative stability.

The thermo-oxidative stability of native and maleated lignins was evaluated by isothermal TGA at 200 °C under an oxygen atmosphere. Since no distinct oxidation induction plateau was observed after oxygen introduction, as shown in Figure 12, the conventional oxidation induction time (OIT) could not be accurately determined from the TGA curves. Therefore, three comparative parameters were employed to assess thermo-oxidative stability: (i) the time required to reach 2% mass loss after the purge gas was switched from nitrogen to oxygen (T_2_,O_2_), (ii) the weight loss after 60 min of oxygen exposure (ΔW_60_), and (iii) the residual mass after 60 min of oxygen exposure, as summarized in Table 4. The T_2_,O_2_ value was calculated from the moment the purge gas was switched from nitrogen to oxygen; therefore, the initial heating and equilibration period under nitrogen was excluded from the calculation. The ΔW_60_ value represents the percentage weight loss after 60 min of oxygen exposure relative to the sample mass at the time of oxygen introduction, whereas the residual mass after 60 min of oxygen exposure represents the remaining sample mass expressed as a percentage of the initial sample mass. These parameters were used to comparatively evaluate the thermo-oxidative stability of the lignin samples when a distinct oxidation induction period could not be identified from the TGA curves [49,50].

Thermo-oxidative degradation of lignin involves oxygen uptake, free-radical generation, hydroperoxide formation, and subsequent oxidative chain reactions leading to mass loss [51]. Therefore, longer oxidation induction times and lower weight losses generally indicate superior oxidative stability. Among all samples, CNF1 exhibited an oxidation induction time of 10.58 min, reflecting the inherent antioxidant activity of lignin arising from its abundant phenolic hydroxyl groups. Phenolic structures can donate hydrogen atoms to stabilize free radicals and terminate oxidation reactions [21,46]. The thermo-oxidative behavior changed considerably after maleation treatment. Samples 5MA160 and 10MA160 exhibited significantly shorter oxidation induction times of 7.29 and 6.59 min, respectively, indicating reduced resistance toward oxidative degradation. This decrease is consistent with the esterification reaction, which consumes phenolic and aliphatic hydroxyl groups and consequently reduces the radical-scavenging capacity of lignin. The reduction in antioxidant activity observed previously from the FRAP analysis further supports this interpretation. Interestingly, samples prepared at 180 °C demonstrated markedly improved oxidative stability. The T_2_,O_2_ values increased to 10.29 min for 5MA180 and 12.55 min for 10MA180, the latter being the highest among all samples. This result suggests that the higher reaction temperature promoted structural rearrangement and condensation reactions within the lignin matrix, leading to a more oxidation-resistant structure. Similar behavior has been reported for thermally treated lignins, where increased condensation reduces the accessibility of oxygen-sensitive sites and enhances oxidative resistance [21,52]. The weight loss after 60 min oxidation (ΔW_60_) further confirmed these observations. Samples 5MA160 and 10MA160 exhibited the highest mass losses (6.03 and 6.30%, respectively), whereas 10MA180 showed the lowest value (4.09%). Correspondingly, the residual mass after 60 min exposure to oxygen was highest for 10MA180 (84.90%) and 5MA180 (84.71%), compared with 83.59% for CNF1. These findings indicate that oxidation proceeded more slowly in the samples modified at 180 °C. The superior thermo-oxidative stability of 10MA180 is particularly noteworthy because it exhibited the highest degree of esterification while retaining the longest oxidation induction time. This suggests that thermo-oxidative stability was governed not only by the concentration of phenolic hydroxyl groups but also by structural factors such as molecular condensation, crosslink density, and oxygen diffusion resistance. Therefore, although esterification reduces antioxidant activity by consuming hydroxyl groups, the formation of a more condensed aromatic network at elevated reaction temperature can compensate for this effect and ultimately improve resistance to oxidative degradation.

#### 2.3.8. Structure–Property Relationship

The combined results obtained from FTIR, degree of esterification analysis, TEM, WAXD, FRAP, thermogravimetric analysis, and thermo-oxidative stability measurements provide a comprehensive understanding of how structural modification influences the properties of maleated nanolignin. FTIR spectroscopy confirmed the successful esterification of CNF1 through the formation of ester carbonyl groups at approximately 1720 cm^−1^ and the progressive increase in the degree of esterification with increasing reaction temperature and maleic anhydride loading. The introduction of maleate functionalities altered intermolecular interactions within the lignin structure, leading to noticeable changes in nanoparticle morphology. TEM analysis showed that the particle size increased from 2.76 ± 0.96 nm for CNF1 to 6.74–9.93 nm after modification. This increase is attributed to changes in molecular packing and self-assembly behavior caused by the incorporation of ester groups and the reduction of intermolecular hydrogen bonding.

The structural modification strongly affected the antioxidant properties of lignin. A clear inverse relationship was observed between degree of esterification and FRAP activity. Native CNF1 exhibited the highest antioxidant activity (47,670 ± 5888 µmol TE/g), whereas the highly esterified sample 10MA180 showed the lowest value (4267 ± 672 µmol TE/g). This behavior is primarily associated with the consumption and masking of phenolic hydroxyl groups during esterification, which reduced the availability of electron-donating sites responsible for radical scavenging and ferric ion reduction.

Thermal analysis further demonstrated that esterification altered the degradation behavior of lignin. Although the incorporation of ester groups introduced thermally labile aliphatic structures that reduced T_max_ and char residue, several modified samples exhibited higher T10% values than native CNF1, indicating improved resistance to the initial stages of thermal degradation. WAXD analysis revealed that both native and modified lignins remained predominantly amorphous, suggesting that the observed property changes originated mainly from chemical modification rather than crystallinity development. Interestingly, the thermo-oxidative stability results showed a trend opposite to that observed for antioxidant activity. While FRAP activity decreased with increasing esterification degree, oxidation resistance improved under severe modification conditions. The highly esterified sample 10MA180 exhibited the highest T_2_,O_2_ value (12.55 min), the lowest weight loss after 60 min oxidation (ΔW_60_ = 4.09%), and the highest residual mass after oxidation (84.90%). These results suggest that the introduction of maleate functionalities and possible condensation reactions occurring at elevated reaction temperatures generated a structure that was less susceptible to oxidative degradation. In contrast, samples modified under milder conditions (5MA160 and 10MA160) exhibited lower T_2_,O_2_ values and greater oxidative weight loss, indicating lower oxidation resistance. The degree of esterification emerged as the key parameter governing the physicochemical performance of maleated nanolignin. Increasing esterification enhanced chemical functionality and thermo-oxidative stability but simultaneously reduced antioxidant activity and char-forming ability. These findings demonstrate a clear structure–property relationship and provide important guidelines for tailoring lignin-based nanomaterials toward specific applications. Native or lightly modified lignins may be preferred for antioxidant applications, whereas highly esterified lignins such as 10MA180 are more suitable for bio-based resins, coatings, and thermally durable materials requiring enhanced oxidative stability.

## 3. Materials and Methods

### 3.1. Materials and Reagents

Coconut husk was obtained from Chonburi province, Thailand. Sodium hydroxide (NaOH), ethanol (EtOH, 95%), sulfuric acid (H_2_SO_4_), sodium acetate trihydrate and acetic acid were purchased from QRëC^TM^, Auckland, New Zealand. Maleic anhydride (MA), 2,4,6-tripyidyl-s-triazine and Trolox standard were purchased from Tokyo Chemical Industry, Tokyo, Japan. Dimethyl sulfoxide (DMSO) and ferric chloride (FeCl_3_) were purchased from Loba Chemie, Maharashtra, India. All chemicals were used as received without further purification.

### 3.2. Preparation and Fractionation of Nanolignin

Coconut husk lignin was extracted by alkaline treatment following a modified procedure. Briefly, coconut husk powder was mixed with 3 M sodium hydroxide solution at a solid-to-liquid ratio of 1:30 (*w*/*v*) and subjected to ultrasonic-assisted extraction at 80 °C for 4 h. The mixture was then cooled and allowed to stand at room temperature for 24 h. The suspension was filtered under vacuum to remove insoluble residues. The filtrate was acidified with H_2_SO_4_ to pH 2, resulting in lignin precipitation. The precipitated lignin was collected by vacuum filtration, thoroughly washed with distilled water and dried at 50 °C for 48 h.

The obtained lignin (CNL) was subsequently fractionated based on ethanol solubility. First, the dried lignin was dispersed in 95% EtOH and sonicated at room temperature for 60 min. The mixture was separated by vacuum filtration into soluble and insoluble fractions. The solvent from the ethanol-soluble fraction was removed by rotary evaporation, and the recovered fraction was designated as CNF1.

The insoluble residue obtained from the 95% ethanol treatment was further dispersed in 80% ethanol and sonicated at room temperature for 60 min. After vacuum filtration, the ethanol-soluble fraction was collected, and the solvent was removed by evaporation to obtain CNF2. The insoluble residue remaining after extraction with both 95% and 80% ethanol was dried and designated as CNF3. The resulting fractions (CNF1, CNF2, and CNF3) were stored in sealed containers and subsequently characterized for their structural, morphological, thermal, and antibacterial properties.

### 3.3. Synthesis of Maleated Nanolignin

Maleated nanolignin was synthesized via microwave-assisted esterification of CNF1 with maleic anhydride. Briefly, CNF1 and maleic anhydride were weighed according to the desired weight ratio and transferred into a Teflon digestion vessel. DMSO was then added as a reaction medium, and the mixture was stirred until a homogeneous solution was obtained. The reaction vessel was sealed with a pressure-resistant safety cap and subjected to microwave irradiation using a microwave digestion system (Ethos, Milestone, Italy) at the designated reaction temperature for 5–10 min. After completion of the reaction, the reaction mixture was cooled to room temperature. The modified lignin was subsequently precipitated by the dropwise addition of 40 mL of deionized water under continuous stirring for 30 min. The suspension was then allowed to stand to ensure complete precipitation of the product.

The precipitated maleated nanolignin was recovered by vacuum filtration, thoroughly washed with DI water to remove unreacted reagents and residual solvent, and dried in an oven at 100 °C until constant weight. The dried products were designated according to the maleic anhydride-to-lignin ratio and reaction temperature and subsequently characterized for their structural, morphological, thermal, antioxidant, and thermo-oxidative properties.

The initial optimization experiments were conducted as screening studies to identify the most suitable reaction conditions for microwave-assisted esterification. Based on the screening results, the selected conditions (160 and 180 °C) were subsequently repeated to confirm reproducibility and were then used for all subsequent characterization and property evaluation.

### 3.4. Characterizations

The structural characteristics of CNF1 and maleated lignins were analyzed using attenuated total reflectance Fourier transform infrared (ATR-FTIR) spectroscopy with a PerkinElmer System 2000 spectrometer (PerkinElmer Inc., Waltham, MA, USA). Spectra were recorded over the wavenumber range of 4000–400 cm^−1^ at a resolution of 4 cm^−1^, with four scans accumulated for each measurement.

The occurrence of esterification and the relative extent of structural modification were evaluated from the FTIR spectra. Particular attention was given to the absorption band at approximately 1720 cm^−1^, which is attributed to the stretching vibration of ester carbonyl groups (C=O) formed during the reaction between lignin hydroxyl groups and maleic anhydride. To estimate the relative degree of esterification, the peak height (absorbance intensity) of the carbonyl band at 1720 cm^−1^ (I_1720_) was normalized against the peak height of the aromatic skeletal vibration band at 1510 cm^−1^ (I_1510_). The band at 1510 cm^−1^ was selected as an internal reference because the aromatic backbone of lignin remains largely unchanged during esterification. The relative degree of esterification (I_C=O_) was calculated according to Equation (1) [17,43]:

(1) $$I_{C=O}=\frac{I_{1720}}{I_{1510}}$$

The morphological characteristics of CNF1 and maleated lignin samples were investigated using using a Tecnai 20 transmission electron microscope (TEM) (Philips/FEI, Eindhoven, The Netherlands) operated at an accelerating voltage of 120 kV. Structural organization and crystallinity were further examined by wide-angle X-ray diffraction (WAXD) using a D2 PHASER X-ray diffractometer (Bruker AXS GmbH, Karlsruhe, Germany). Diffraction patterns were recorded over a 2*θ* range of 5–80° using CuKα radiation with a wavelength of 1.54 Å. All measurements were conducted under standard operating conditions at room temperature.

The antioxidant activity of CNF1 and maleate lignin samples was determined using the Ferric Reducing Antioxidant Power (FRAP) assay following the method of Benzie and Strain [22] with slight modifications. The FRAP reagent was freshly prepared by mixing 0.3 M acetate buffer (pH 3.6), 10 mM 2,4,6-tripyridyl-s-triazine solution in 40 mM HCl, and 20 mM FeCl_3_·6H_2_O at a volumetric ratio of 10:1:1 and pre-incubated at 37 °C. Briefly, 2.0 mL of FRAP reagent was mixed with 100 µL of lignin solution (0.25 mg/mL in EtOH), incubated at 37 °C for 10 min in the dark, and the absorbance was measured at 593 nm using a Cary 3500 UV–Vis spectrophotometer (Agilent Technologies, Santa Clara, CA, USA). Trolox (0–1000 µM) was used to construct a calibration curve, and the results were expressed as micromoles of Trolox equivalents per gram of lignin (µmol TE/g). All measurements were performed in triplicate, and the data were reported as mean ± standard deviation.

The thermal stability of the samples was evaluated using a TA Q500 thermogravimetric analyzer (TA Instruments, New Castle, DE, USA) under a nitrogen atmosphere. Thermogravimetric measurements were performed from 40 to 800 °C at a heating rate of 10 °C min^−1^. Thermo-oxidative stability was investigated by isothermal TGA at 200 °C. The samples were initially heated and equilibrated under nitrogen, after which the purge gas was switched to oxygen. The oxidation process was monitored for 60 min, and the oxidation induction time (OIT) together with the residual mass after 60 min of oxidation (ΔW_60_) were used as indicators of thermo-oxidative resistance.

Antibacterial activity test of lignin was performed. The antibacterial activity of nano-lignin samples was evaluated using the Time-Kill Assay method according to the U.S. Food and Drug Administration (FDA) guideline. Gram-positive bacteria, *Staphylococcus aureus* ATCC 25923, and Gram-negative bacteria, *Escherichia coli* ATCC 25922, were used as the test microorganisms. Briefly, the bacterial cultures were first grown in Tryptic Soy Broth (TSB) for 4–6 h to obtain cells in the logarithmic growth phase. The bacterial suspensions were then diluted with sterile 0.85% NaCl solution to achieve a turbidity equivalent to McFarland standard No. 0.5, corresponding to approximately 1.0 × 10^8^ CFU/mL. Subsequently, 1 mL of bacterial suspension was added into sterile test tubes containing the nano-lignin samples. After 24 h of incubation, the surviving bacterial cells were quantified using the spread plate technique. Serial dilutions of the bacterial suspensions were prepared from 10^−1^ to 10^−8^ using sterile diluent. Aliquots (100 µL) from each dilution were spread onto plate count agar plates in triplicate under aseptic conditions. The inoculated plates were incubated at 37 °C for 24 h, after which the bacterial colonies were counted and expressed as colony-forming units per milliliter (CFU/mL). The antibacterial activity efficiency (AAE) was calculated based on the reduction in viable bacterial counts by comparing the initial bacterial population (N_0_) with the surviving bacterial population after treatment (N_s_), according to Equation (2):

(2) $$AAE\left(\%\right)=\frac{N_{0}-N_{s}}{N_{0}}\times 100$$

## 4. Conclusions

Coconut husk lignin was successfully fractionated into nanolignin fractions with distinct physicochemical properties. Among the fractions obtained, CNF1 exhibited the most favorable combination of nanoparticle size, antibacterial activity, and thermal stability and was therefore selected as the precursor for further chemical modification.

Microwave-assisted esterification with maleic anhydride successfully introduced maleate functionalities into the lignin structure, as confirmed by ATR-FTIR spectroscopy and the degree of esterification analysis. The extent of modification increased with increasing reaction temperature and maleic anhydride loading, reaching the highest degree of esterification for the 10MA180 sample. Esterification altered the self-assembly behavior of nanolignin, resulting in larger nanoparticle sizes while preserving the predominantly amorphous structure of the material.

The structural modification significantly influenced the functional properties of lignin. Antioxidant activity decreased progressively with increasing degree of esterification due to the consumption of phenolic hydroxyl groups, which are primarily responsible for electron donation and radical scavenging activity. In contrast, thermo-oxidative stability improved at higher degrees of esterification. The 10MA180 sample exhibited the highest oxidation resistance, as evidenced by the highest T_2_,O_2_ value (12.55 min), the lowest oxidative weight loss after 60 min (ΔW_60_ = 4.09%), and the highest residual mass after oxidation (84.90%). This research demonstrates that the degree of esterification is a key structural parameter governing the morphology, antioxidant performance, thermal behavior, and oxidation resistance of maleated nanolignin. A clear structure–property relationship was established, revealing that increasing esterification decreases antioxidant activity but enhances thermo-oxidative stability. These findings provide valuable guidance for the future design of lignin-based nanomaterials for applications ranging from antioxidant additives to bio-based resins, coatings, and thermally durable sustainable materials.

Future studies will focus on the application of maleated nanolignin as a renewable reactive component in bio-based alkyd resins, protective coatings, and sustainable composite materials. Further investigation of interfacial adhesion, mechanical performance, and long-term thermo-oxidative durability is expected to facilitate the development of high-performance lignin-based materials with reduced dependence on petroleum-derived resources.

## Figures and Tables

**Table 1:** Antibacterial activity of lignin fractions.

Sample	*Staphylococcus aureus*		*Escherichia coli*	
	Ns (CFU/mL)	AAE (%)	Ns (CFU/mL)	AAE (%)
CNL	2.40 × 10^2^	100	2.26 × 10^8^	0
CNF1	1.00 × 10^0^	100	2.33 × 10^8^	0
CNF2	1.00 × 10^0^	100	2.44 × 10^8^	0
CNF3	1.52 × 10^5^	100	2.44 × 10^8^	0
N_0_	1.93 × 10^8^		1.26 × 10^8^	

**Table 2:** Experimental conditions, product yields and degree of esterification for microwave-assisted esterification of CNF1 with maleic anhydride.

Sample	Temperature (°C)	Time (min)	Lignin:MA	Yield (%)	I_C=O_
10MA80	80	10	1:10	43.0	0.9
10MA100	100	10	1:10	45.0	1.1
10MA120	120	10	1:10	43.1	1.6
10MA140	140	10	1:10	52.4	2.1
0MA160	160	10	1:0	0.0	NA
2.5MA160	160	10	1:2.5	62.3	1.4
5MA160	160	10	1:5	87.0	2.9
10MA160	160	10	1:10	88.1	5.0
15MA160	160	10	1:15	66.7	3.0
5MA180	180	10	1:5	96.9	6.0
10MA180	180	10	1:10	108.5	13.0

**Table 3:** Characteristic thermal degradation temperatures and char residue of CNF1 and maleated lignins obtained from TGA analysis.

Sample	T10% (°C)	T20% (°C)	T_max_ (°C)	Residue at 600 °C (%)
CNF1	186.22	283.45	326.16	45.38
5MA160	191.21	269.36	309.72	26.75
5MA180	205.62	286.11	310.38	29.86
10MA160	183.64	261.86	309.56	25.81
10MA180	194.71	287.64	320.75	24.22

**Table 4:** Thermo-oxidative stability parameters of CNF1 and maleated lignins.

Sample	T_2_,O_2_ (min)	ΔW_60_ (%)	Residual Mass After 60 min O_2_ (%)
CNF1	10.58	4.54	83.59
5MA160	7.29	6.03	81.91
5MA180	10.29	4.64	84.71
10MA160	6.59	6.30	80.71
10MA180	12.55	4.09	84.90

## Data Availability

The data supporting these conclusions of the article is available upon request.
